# Poly (red DSBR)/Al-ZnO modified carbon paste electrode sensor for dopamine: a voltammetric study

**DOI:** 10.1038/s41598-021-93723-6

**Published:** 2021-07-12

**Authors:** J. K. Shashikumara, B. E. Kumara Swamy, S. C. Sharma, S. A. Hariprasad, Kaustubha Mohanty

**Affiliations:** 1 Department of P.G. Studies and Research in Industrial Chemistry, Kuvempu University, Jnanasahyadri, Shankaraghatta, Shivamogga, Karnataka 577451 India; 2 Director, National Assessment and Accreditation Council, (Work carried out as Honorary Professor, Jain University), Bangalore, Karnataka 560 069 India; 3 School of Energy Science and Engineering, Indian Institute of Technology Guwahati, Guwahati, 781039 India; 4 Jain Deemed-to-be University, Bangalore, Karnataka 560 069 India; 5 Department of Chemical Engineering, Indian Institute of Technology Guwahati, Guwahati, 781039 India

**Keywords:** Chemistry, Materials science, Physics

## Abstract

In the present work, the ZnO and Al-ZnO nanoflakes (NFs) were synthesized by the co-precipitation process. The synthesized NFs were characterized by X-ray diffraction and field emission scanning electron microscopy. Energy dispersive X-ray spectrometer was explored for the elemental chemical compositions. The prepared NFs were taken for the modification of the electrode and developed the modified electrode for the electrochemical analysis of the dopamine (DOA) at pH 7.4. The Al-ZnO modified carbon paste electrode (MCPE) was electropolymerised by using textile dye red DSBR. The Po-RD/Al-ZnO MCPE exhibited good electrochemical sensor properties towards the electrochemical detection of DOA. Several factors such as the impact of speed rate (υ), pH and concentration of the DOA were analyzed at the modified electrode. The great sensitivity was established to the fast electron-transfer kinetics and surface coverage of the DOA on the electrode. The prepared electrode exhibits satisfactory stability at the ambient conditions. The detection limit of 0.58 μM was achieved for the DOA. The decorated sensor was stable, sensitive, selective, and reproducible and used in the analytical applications.

## Introduction

Now-a-days, several NFs have been developed and they show good active properties for the electrochemical studies. The metal oxide NFs have fabulous attention because of its easily-obtained and favorable sensing properties, specific surface areas, optimum optical and electronic properties, outstanding catalytic activities, great conductivities^1–5^. Zinc oxide (ZnO) is a massively intended nano-structured metal oxide and is an impressively inspected scientific inorganic material because of its special semiconducting and exclusive sensing properties to sense the various gases and vapors. It is additionally used in several fields of applications such as gas sensors, optoelectronic devices, variations, and transparent conducting electrodes. Now-a-days, numerous researchers are checking ZnO NFs to execute multidimensional phenomenal research and also to use it as a modifier due to its astounding surface properties like sensing, electronic, and catalytic properties^6–11^. Doping is one of the good and effective methods used to increase conductivity and it improves the sensing properties of metal-oxide semiconductors by altering the energy-band structure and morphology because dopants can afford electronic defects. A number of reports are obtainable on the improvement of the sensing properties of ZnO by adding different doping materials. The ‘Al’ is considered a promising dopant and shows low resistivity in the thin-film shape. Also, Al-doped ZnO thin films are of specific interest because of their less price of raw material, good electrical and optical properties for the applications in light-emitting diodes, organic sensors, mass production of photovoltaic devices, and liquid crystal display^12–18^.

Carbon paste electrodes (CPEs) have interesting advantages such as reproducibility, stability, and renewability of the surface which makes them one of the most attractive materials as working electrodes^19,20^. To date CPEs more applicable in wide areas such as pharmacological, biological, and environmental analysis, due to their low price when compared to other materials. CPE’s electrochemical properties such as adsorption capacity, selectivity, and sensitivity can be enhanced by physical or chemical medications^21,22^. The reason to modify a CPE matrix is to obtain a new sensor with desired electrochemical properties.

DOA is the tremendously crucial to associative neuron communication in the human central nervous system as a chemical messenger. The deviation of DOA concentration in the human body may cause neurological disorders such as Parkinson’s disease. Diseases are distorted the lives of people around the world through their deficiency of DOA in the human body for that reason developing a sensor for DOA is very significant^23–26^.

In this work, ZnO and Al-ZnO NFs were synthesized by the co-precipitation method, the formation of the NFs were confirmed by XRD and FESEM with EDX. The synthesized NFs were used as the modifiers and Al-ZnO NFs were electropolymerised by Red DSBR. Finally, a poly (Red DSBR)/Al-ZnO modified Carbon Paste electrode (Po-RD/Al-ZnO MCPE) was developed. The developed electrode displayed good electrocatalytic activity towards DOA. The various parameters such as the effect of scan rate, pH and concentration of DOA were studied. The sensor can be used for the DOA assay in pharmaceutical samples.

## Experimental segment

### Materials selection and apparatus

Dopamine hydrochloride, uric acid (UA), ascorbic acid (AA), folic acid (FA), sodium hydroxide, Na_2_HPO_4,_ zinc acetate hexahydrate [Zn(CH_3_COO)_2_·6H_2_O)], aluminum acetate hexahydrate [Al(CH_3_COO)_2_·6H_2_O], NaH_2_PO_4_ and ethanol were procured from Nice Chemicals Pvt. Ltd. (India). The silicon oil (Himedia), graphite powder (Loba Chemie) and Red DSBR (Astik Dyestuff Pvt. Ltd) were used directly as received along with other chemicals as listed above. Double distilled water was used as solvent throughout the experimental procedure. Structural analyses of the films were captured by FESEM and elemental chemical compositions were found out from EDX. The electrochemical research was carried on a voltammetric instrument of model CH Instrument-CHI 660c electrochemical workstation.

### Preparation of ZnO and Al-ZnO NFs

**Figure 1:**
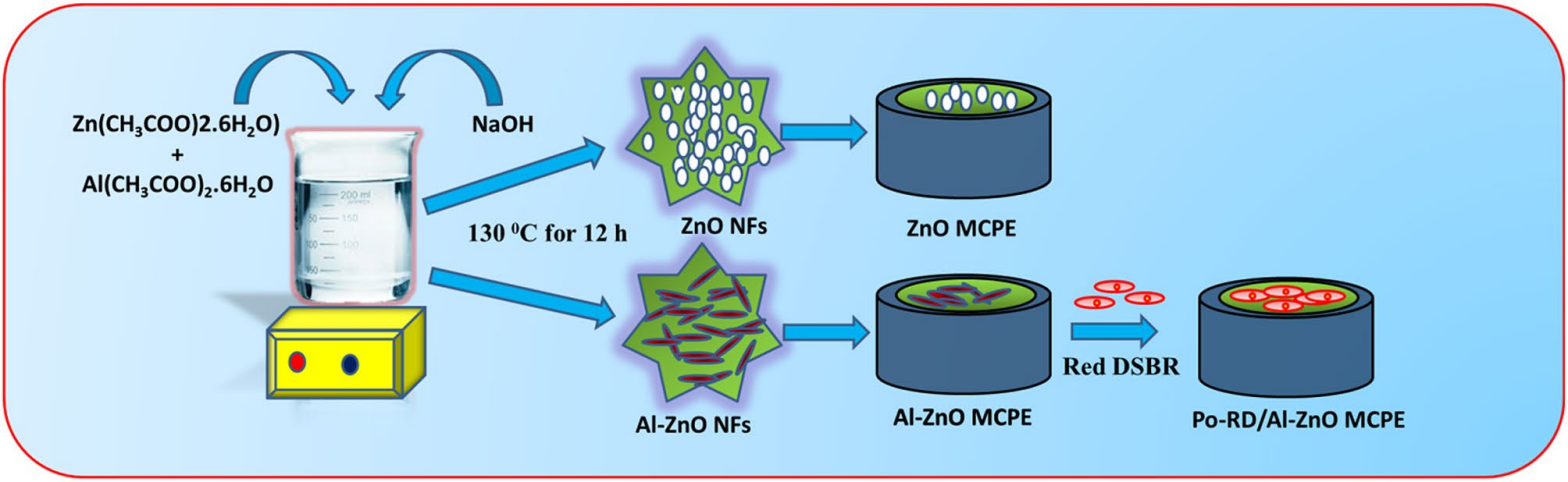
Schematic representation of the stepwise synthesis of NFs and fabrication of electrode.

ZnO and 5% Al-doped ZnO were synthesized by the co-precipitation technique. The host precursor was zinc acetate hexahydrate while doping one was the aluminum acetate hexahydrate. For synthesizing the undoped ZnO NFs, a suitable quantity of Zn(CH_3_COO)_2_ was dissolved in 1:1 ratio of ethanol to water system under magnetic stirring for 20 min at the room temperature. Then, an aqueous NaOH solution was added to the above solution until the pH value reached 7.0. The resultant solution was continuously stirred for 2 h under the magnetic stirring. The precipitate obtained was centrifuged at 5000 rpm for 4 min and washed many times with ethanol. The white solid was dried in a hot air oven at 130 °C for 12 h. The obtained powder was then calcinated for 5 h at 600 °C in a muffle furnace. The Al-doped ZnO NFs were synthesized using the same process with 5% Al precursor (Fig. 1).

### Setting up of the electrodes

**Figure 2:**
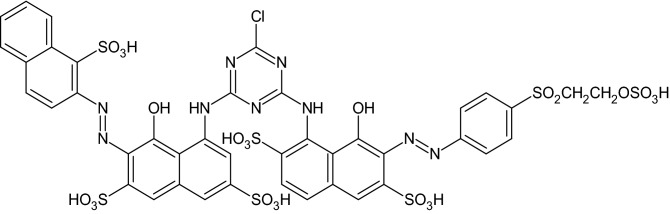
Structure of Red DSBR.

The CPE was modified by taking 4 mg ZnO and Al-ZnO NFs in silicon oil and graphite powder (70:30 w/w). Then this mixture was systematically mixed in an agate mortar for about 30 min and filled into a homemade Teflon cavity and polished by the soft paper^27^. Without NFs process is exactly similar to the bare carbon paste electrode (BCPE). The composite Po-RD/Al-ZnO MCPE developed by Al-ZnO MCPE was again fabricated by the electrodeposition of 1 mM aqueous Red DSBR monomer (Fig. 2) accommodating with 0.1 M NaOH within the range of − 0.8 to 1.1 V at the speed rate of 100 mV s^−1^ and then the electrode was cleaned with double distilled water^28^.

## Outcome and discussion

### XRD analysis

**Figure 3:**
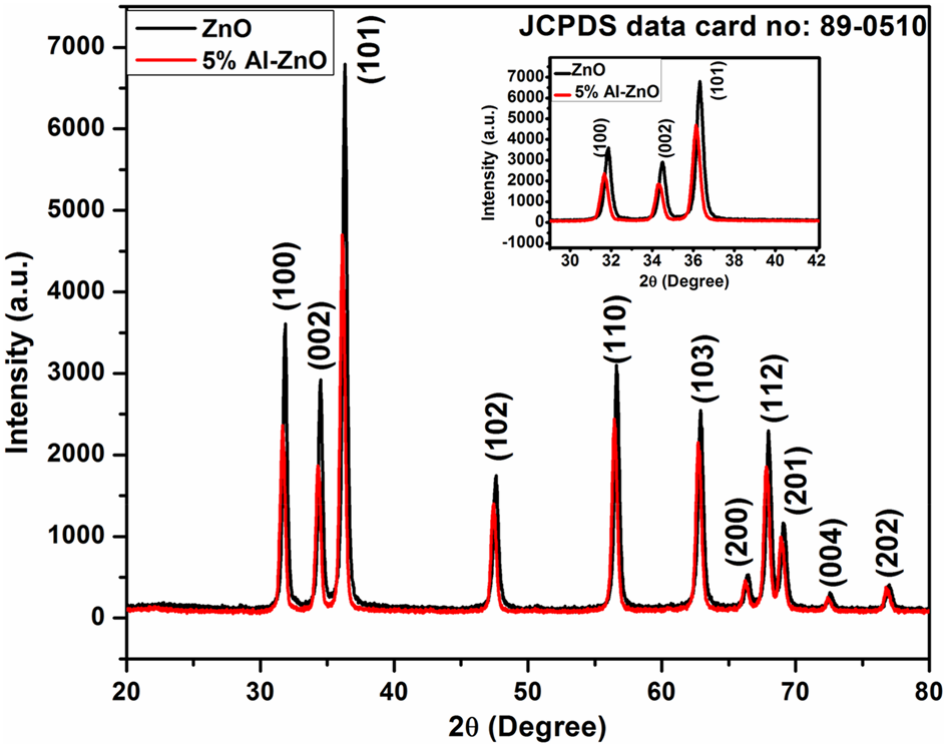
XRD patterns of ZnO and 5% Al-ZnO NFs. Inset shows the peak shifting character of the plane (100) (002) and (101) towards lower angle for Al-ZnO.

Figure 3 displays the XRD arrangements of the synthesized ZnO and Al-ZnO NFs. The sharp distinctive peaks were observed for both samples in the arrangements corresponding the perfect wurtzite structure of ZnO (JCPDS data card no: 89-0510). The achieved phase was pure and no peak was observed correspond to Al indicates that the Al atoms were effectively corroborate into ZnO lattice. Inset Fig. 3 displays small switch of the peaks towards lower 2θ value because of Al content. The dissimilarity in the ions size cause local distortion of the lattice due to specific residual stress inside the NFs^29^. Moreover intensity of all peaks reduced with doping indicated the decrease in the crystallinity of ZnO NFs because of the corroboration of defects in the lattice site^30^. Scherrer’s Eq. (1)^31^ was employed to calculate the average crystallite size of the ZnO and Al-ZnO NFs.

1 $$D=\frac{k\lambda }{{\beta }_{hkl}\mathrm{c}\mathrm{o}\mathrm{s}\theta }$$

where ‘k’ was a constant of 0.94, ‘λ’ was the X-ray wavelength of 1.54060 Å for Cu-Kα, θ was the Bragg diffraction angle, and ‘β’ was the full width half maxima. The calculated crystallite size of the NFs is presented in Table 1. It is evident from Table 1 that the increase of Al content resulted in decrease of average crystallite size. The reduction in the crystallite size was mainly due to distortion in the host lattice by the foreign impurities (i.e., Al^3+^) that decrease the nucleation and subsequent growth rate of ZnO NFs. Such type of decrease in crystallite size with increasing in Al dopant level in ZnO lattice was also reported by Zamiri et al*.*^32^.

**Table 1:** Structural parameter derived from XRD data**.**

Sample	2 θ (^0^)	FWHM	Crystallite size	Micro-strain (ε)	Lattice parameters	
					a	c
ZnO	36.4101	0.2841	30.76	0.0038	3.2501	5.2113
Al-ZnO	36.3473	0.3368	25.93	0.0045	3.2523	5.2193

### Morphological characterization

**Figure 4:**
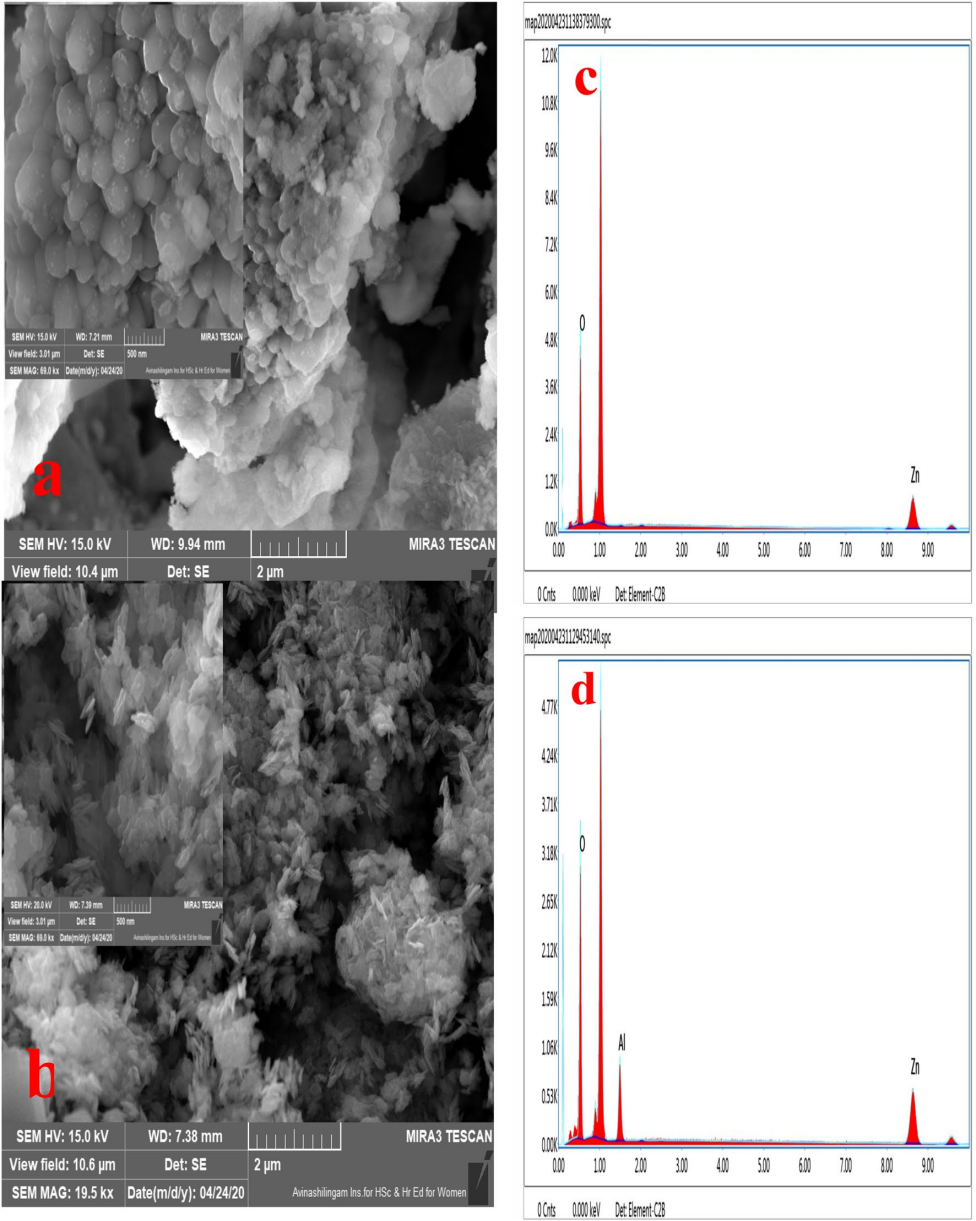
FESEM images of (**a**) ZnO NFs, (**b**) 5% Al-ZnO NFs, (**c**) EDX of ZnO NFs, (**d**) EDX of 5% Al NFs.

FESEM picture of the pure and Al-doped ZnO are depicted in Fig. 4a,b. It is exposed the surface of pure ZnO appearances like a cluster of spherical nanoparticles. Interestingly, after 5% Al doping, the cluster of spherical nanoparticles of ZnO converted into NFs-like construction. Figure 4c,d shows the EDXA spectra of the pure and Al-doped ZnO NFs. The appearance of well-defined peaks associated to Zn, O, and Al approves the effective doping of Al into ZnO.

### Electrochemical characterization

**Figure 5:**
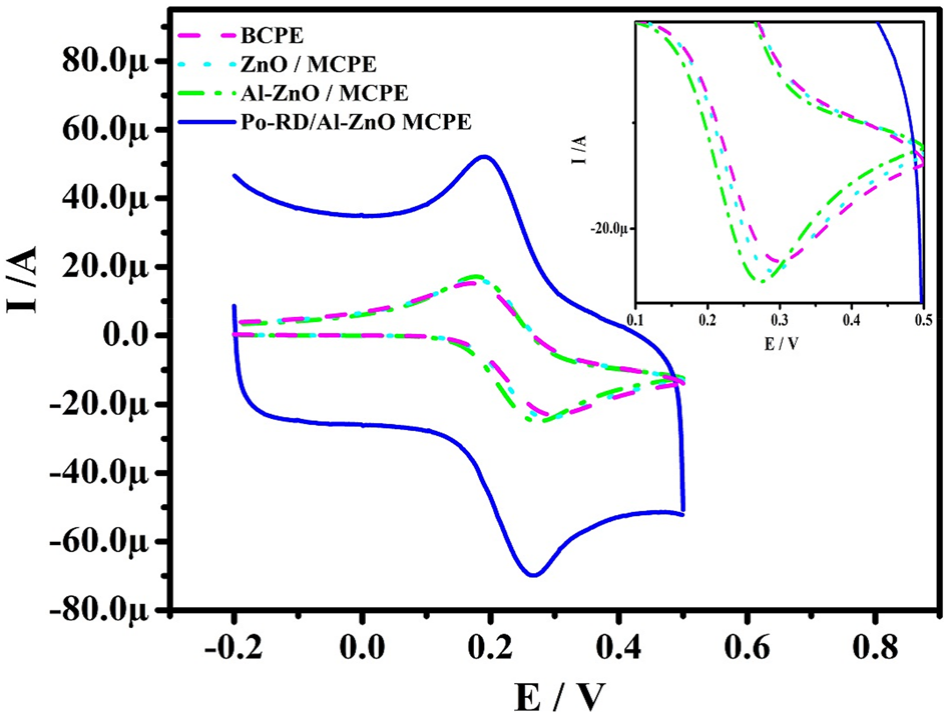
CVs of 1 mM potassium ferrocyanide in 1 M KCl at speed rate 50 mV s^−1^.

Figure 5 depicts the CV curve developed at a speed rate of 50 mV s^−1^ for MCPEs and BCPE disperse in 1 M KCl containing 1 mM K_4_ [Fe (CN)_6_] electrolyte. The electrode active surface area for the reversible reaction was calculated by accepting Randles–Sevcik Eq. (2)

2 $${\text{I}}_{{\text{p}}} = \left( {{\text{2}}.{\text{69 }} \times {\text{ 1}}0^{{\text{5}}} } \right){\text{n}}^{{{\text{3}}/{\text{2}}}} {\text{AD}}_{0} ^{{{\text{1}}/{\text{2}}}} {\text{C}}_{0} \nu ^{{{\text{1}}/{\text{2}}}}$$

where Ip is the peak current, υ and A are the scan rate and area of the working electrode respectively, n is the number of electrons exchanged. The Po-RD/Al-ZnO MCPE exhibits excellent current response and acceptably redox peak potential separation (ΔE) decreases for Po-RD/Al-ZnO, Al-ZnO, and ZnO MCPEs compared to BCPE and also active surface area increased for MCPEs results presented in Table 2. Hence, the result recommends that the Po-RD/Al-ZnO MCPE possessed good electrocatalytic activity.

**Table 2:** Active surface area for electrodes.

Electrodes	ΔE (mV)	Active surface area (cm^2^)
BCPE	129	0.0336
ZnO MCPE	109	0.0382
Al-ZnO MCPE	86	0.0406
Po-RD/Al-ZnO MCPE	74	0.0572

### Electrochemical response for DOA at Po-RD/Al-ZnO MCPE

**Figure 6:**
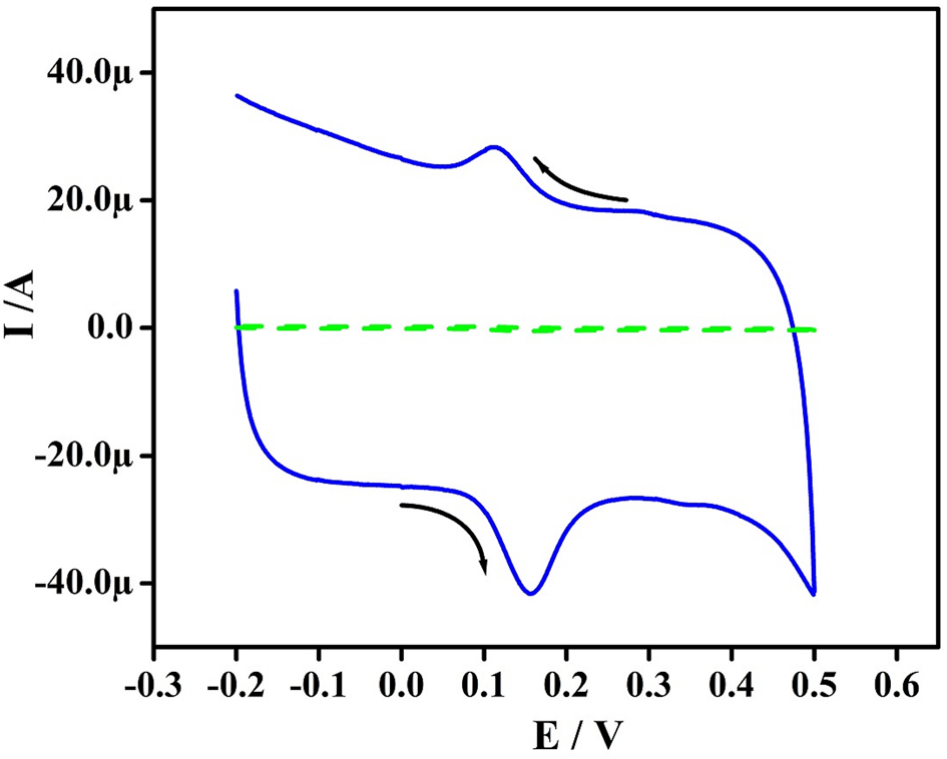
CVs of 5 μM DOA in 0.2 M PBS (pH 7.4) at BCPE and Po-RD/Al-ZnO MCPE at a speed rate of 50 mV s^−1^.

Figure 6 exhibits a CV profile of 5 μM DOA at BCPE & Po-RD/Al-ZnO MCPE in 0.2 M phosphate buffer solution (PBS) (pH 7.4). The Po-RD/Al-ZnO MCPE exhibits hike in redox Ip and a slight shift in the potential but BCPE the DOA exhibits less current with extensive peaks. The obtained results showed that modified electrode exhibits marvelous performance and it decreased the ΔE with enhancement in anodic peak current (Ipa). These results indicate the catalytic effect of the developed sensor on DOA analysis.

### Consequence of speed rate

**Figure 7:**
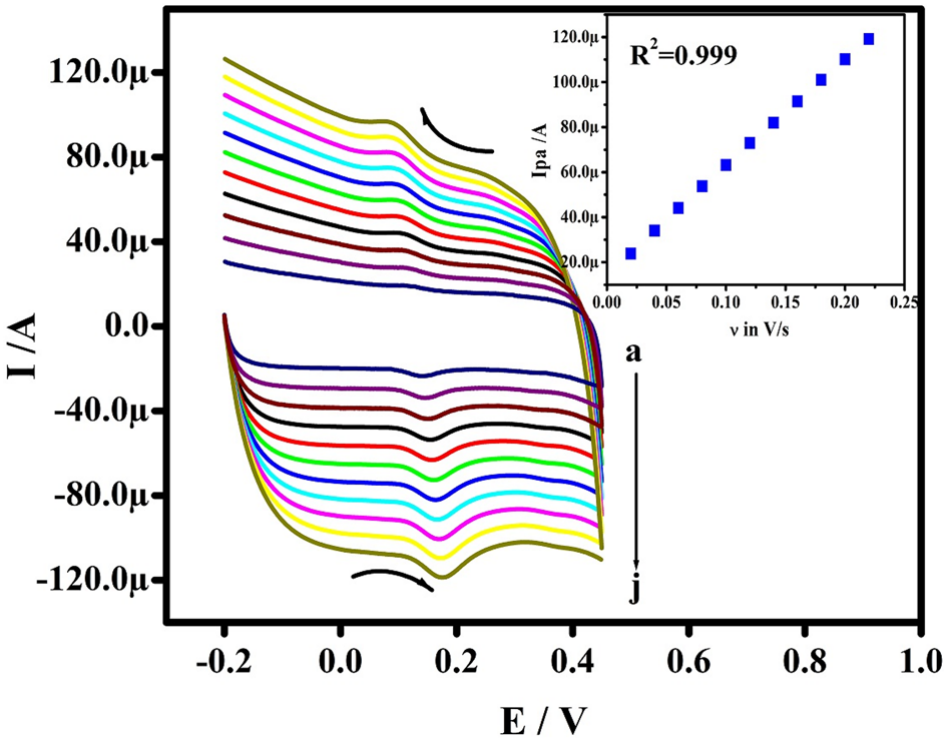
CVs of 5 μM DOA in 0.2 M PBS of pH 7.4 at Po-RD/Al-ZnO MCPE at numerous speed rates (**a**–**j**; 20–200 mV s^−1^). Inset graph is Ipa v/s υ.

The impact of the potential speed rate (υ) on the electrochemical performance of the DOA was studied in PBS (pH 7.4) at Po-RD/Al-ZnO MCPE. When the υ was raised in the range from 20 to 200 mV s^−1^ then the peak current Ip hiked with a slender positive shift in the peak potential as presented in the Fig. 7. The figure of the current intensity variations in terms of speed rates display in inset Fig. 7 and it exhibits good linearity. The results demonstrate that the oxidation–reduction reaction of DOA is under the control of adsorption. The k^0^ values for DOA are recorded in Table 3 and it was carried out by the ΔE from the investigational data and Eq. (3)^33^

3 $$\Delta {\text{Ep}} = {\text{2}}0{\text{1}}.{\text{39 log }}(\nu /{\text{k}}^{0} ) - {\text{3}}0{\text{1}}.{\text{78}}$$

**Table 3:** Heterogeneous rate constant of DOA at different speed rate.

υ/mV/s	ΔEp/mV	k°(s^−1^)
20	29	0.45556
40	37	0.83148
60	43	1.16453
80	50	1.43328
100	57	1.65379
120	60	1.91764
140	63	2.16181
160	67	2.36019
180	89	2.06471
200	81	2.51385

### Repercussion of concentration and influence of solution pH

**Figure 8:**
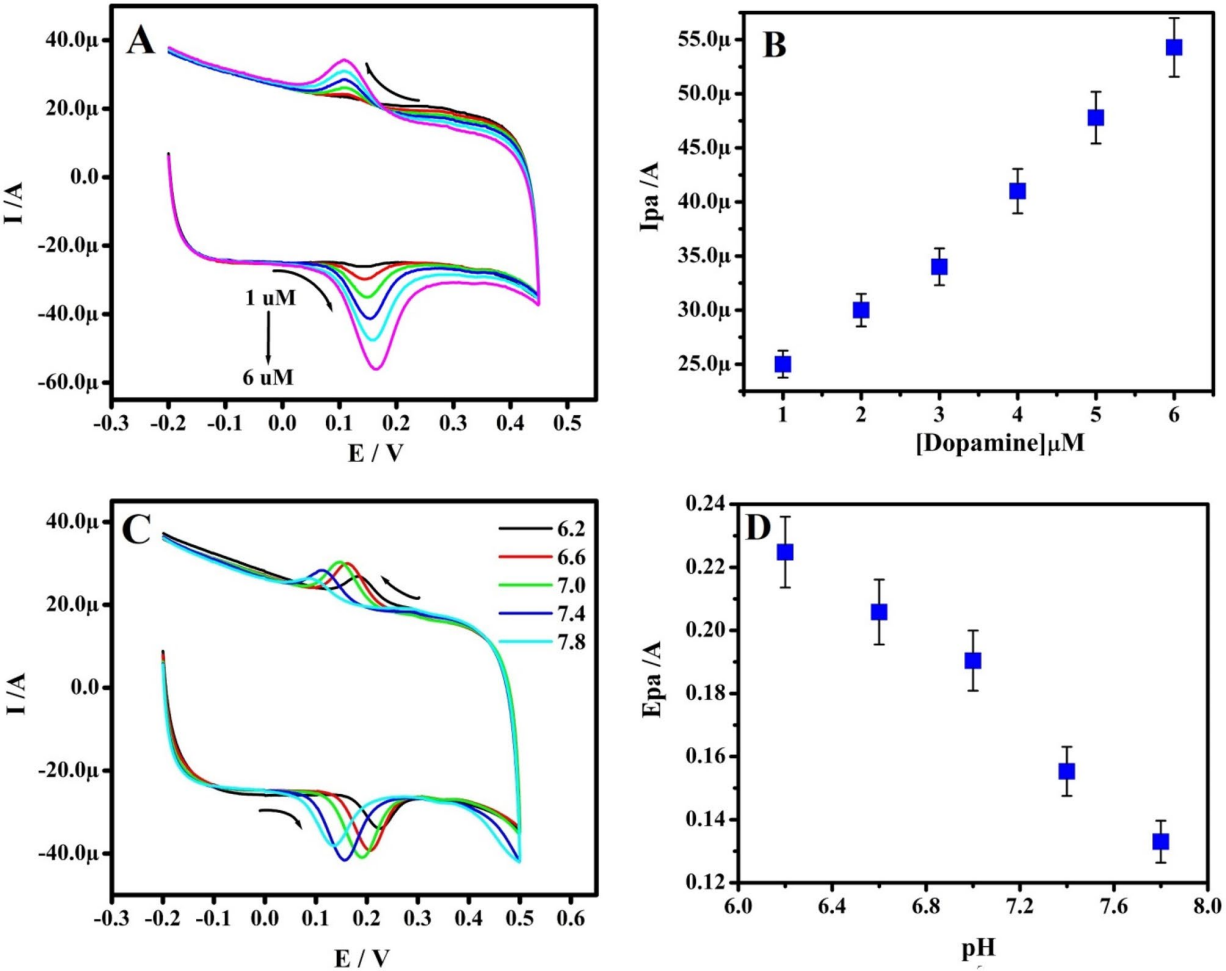
(**A**) CVs of DOA in 0.2 M PBS (pH 7.4) at Po-RD/Al-ZnO MCPE with various concentrations (1–6 μM). (**B**) The plot of Ipa v/s concentration OF DOA. (**C**) CVs for different pH of 5 μM DOA in 0.2 M PBS. (**D**) Plot of Epa v/s pH.

Figure 8A depicted the CV’s of DOA at Po-RD/Al-ZnO MCPE for different concentrations. The concentration of varied beginning in PBS (pH 7.4). The Ip of DOA hiked with hiking the concentration. The design of Ipa versus DOA concentration exhibits good linearity (Fig. 8B) and the lower limit of detection (LLOD) and limit of quantification (LOQ) is determined according to Eqs. (4, 5)^34^ and are presented in Table 4^35–50^.

4 $${\text{LLOD}} = {\text{3 S}}/{\text{M}}$$

5 $${\text{LOQ}} = {\text{1}}0{\text{ S}}/{\text{M}}$$

where S and M specifies the standard deviation and slope.

**Table 4:** Comparative analytical performance electrode for DOA.

Sl. no	Electrodes	LLOD (µM)	Method	References
01	Au/Gr-Au	30	SW	^35^
02	Pt–Au hybrid	24	CV	^36^
03	CTAB/CPE	11.0	DPV	^37^
04	Fc-MCPE	9.4	CV	^38^
05	poly (sudan III)/MCPE	9.3	CV	^39^
06	SWCNT/GCE	7.0	DPV	^40^
07	Metallothioneins self-assembled gold electrode	6.0	CV	^41^
08	LDH/CILE	5.0	DPV	^42^
09	Ag-reduced GO/GCE	5.4	LSV	^43^
10	Poly-VA/MWCNT/GCE	4.5	CV	^44^
11	Ag/Ag2S-CNT-Nafion	4.7	DPV	^45^
12	BPVCM-e/MWCNT/GCE	2.25	CV	^46^
13	Poly(amido black) MCPE	2.03	CV	^47^
14	CTAB-GO/MWNT	1.5	DPV	^48^
15	Au/RGO	1.4	DPV	^49^
16	ERGO	0.5	DPV	^50^
17	Po-RD/Al-ZnO MCPE	0.58	CV	Present work

The CV’s profile for DOA at Po-RD/Al-ZnO MCPE in 0.2 M PBS range 6.2–7.8 portrayed in Fig. 8C. The anodic peak potential (Epa) was lifted towards the negative path with rising in pH and extreme current singles gained at 7.4. The Fig. 8D portrayed the linear establishment between Epa and pH lead to the slope of 58 mV for the DOA. The corresponding pH equation is *E*pa (*V*) =  − 0.0585pH + 0.5915, *R*^2^ = 0.999 (DOA). The achieved slope is nearby to the Nernstiant value for the same number of electrons and protons that are involved in the reactions.

### Success of selectivity and reproducibility

**Figure 9:**
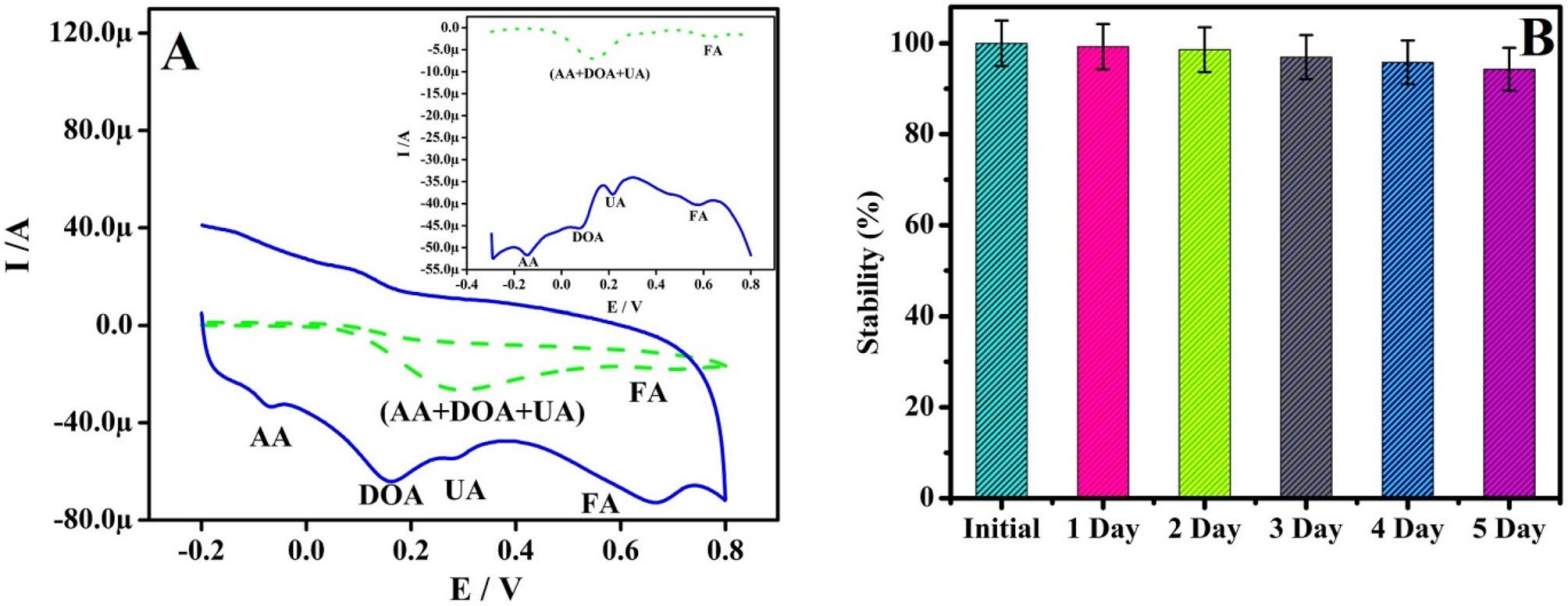
(**A**) CVs for selectivity analysis AA (1 mM), DOA (1 µM), UA (1 µM) and FA (10 µM) at BCPE and Po-RD/Al-ZnO MCPE at a speed rate of 50 mV s^−1^. (**B**) Stability of Po-RD/Al-ZnO MCPE toward DOA at different time intervals.

The CV’s profile for the mixture of Ascorbic acid (AA) (1 mM), DOA (1 µM), Uric acid (UA) (1 µM) and, Folic acid (FA) (10 µM) in 0.2 M PBS (pH 7.4) at BCPE and Po-RD/Al-ZnO MCPE are presented in Fig. 9a. The CVs responses for analytes with short current signals were signifying the poor selectivity and intensity of the BCPE. Nevertheless, Po-RD/Al-ZnO MCPE is the ability to separate the oxidation potential of mixed analytes. This outcome was identifying selectivity Po-RD/Al-ZnO MCPE. The reproducibility of Po-RD/Al-ZnO MCPE was also investigated afterward being kept at the room temperature for 5 days (Fig. 9b). It was found that the current signals recollected 99.25% after first day early current response and the peak potentials were almost unaffected. Likewise second to fifth day current signals almost recollected. This result shows the reproducibility of Po-RD/Al-ZnO MCPE.

### Interference study and analytical application

**Figure 10:**
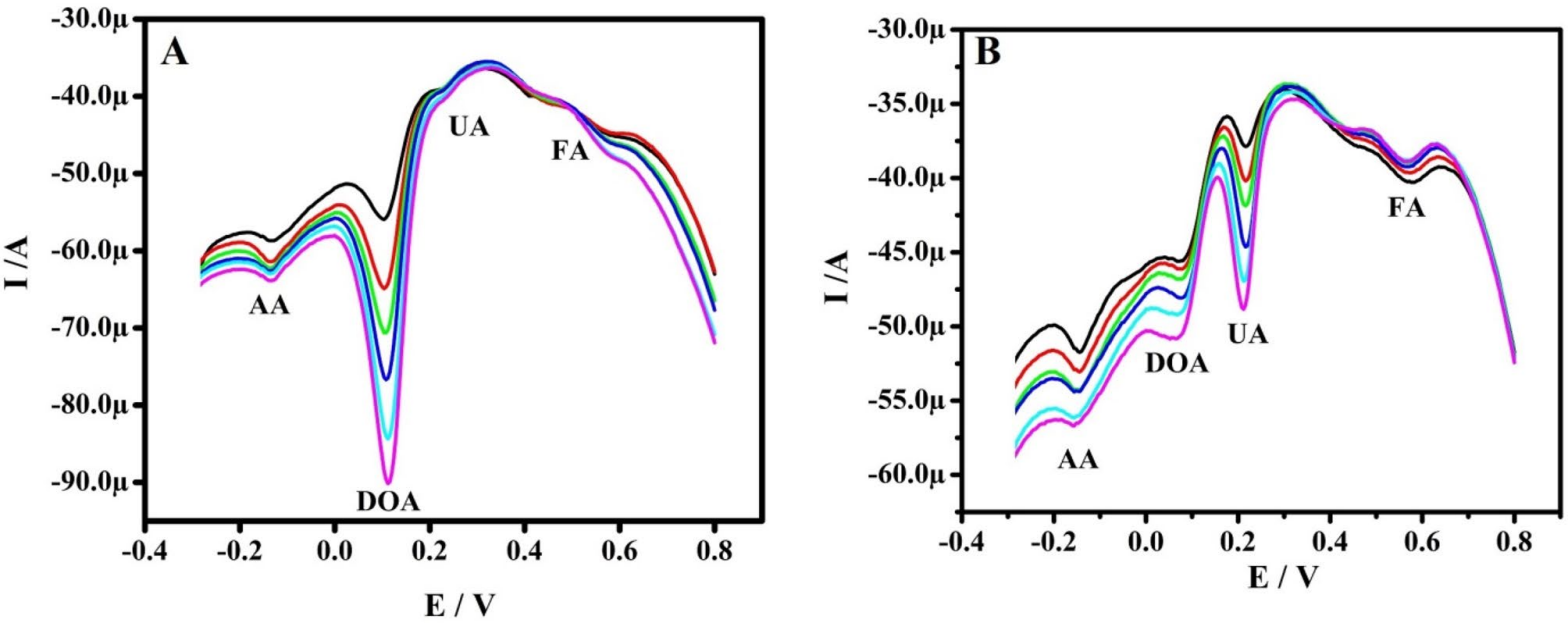
(**A**) DPVs got for different of concentration 1–6 µM DOA in PBS (pH 7.4) with AA (1 mM), UA (1 µM) and FA (10 µM) at Po-RD/Al-ZnO MCPE. (**B**) DPVs got for different of concentration 1–6 µM UA in PBS (pH 7.4) with AA (1 mM), DOA (1 µM) and FA (10 µM) at Po-RD/Al-ZnO MCPE.

The interference study was accomplished in the combination of samples having AA (1 mM), DOA (1 µM), UA(1 µM) and FA (10 µM) at the Po-RD/Al-ZnO MCPE by DPVs. Figure 10a shows that the Ip of DOA was increased with increased concentrations starting 1–6 µM by custody of the constant concentration of AA, UA, and FA. Similarly, UA concentration was varied and its Ip increased with the concentration (Fig. 10b). These consequences showed the stability of Po-RD/Al-ZnO MCPE. The determination of DOA in pharmaceutical sample was studied by CV for practical ability. The Po-RD/Al-ZnO MCPE was performed for the determination of DOA in dopamine hydrochloride injection. The samples were analyzed by standard addition method. The results were found to be satisfactory as revealed from Table 5. This showed a promising application in the biological field.

**Table 5:** Detection of DOA real sample (n = 4).

Sample	Sample added (µM)	Found (µM)	Recovery (%)
DOA	5	4.93	98.64
	10	9.94	99.4
	15	14.87	99.13
	20	19.82	99.1

## Conclusion

In the present work, ZnO and Al-ZnO NFs were synthesized by co-precipitation method and used as the sensor for the DOA. The developed and decorated Po-RD/Al-ZnO MCPE exhibited good current response towards the DOA. The speed rate effect was established as adsorption-controlled electrode process. The pH study exposed participation of equal number of protons and electrons in the redox mechanism. The detection limit of 0.58 μM was achieved for DOA Po-RD/Al-ZnO MCPE can separate the oxidation potential of AA, DOA, UA, and FA with large potential difference compared to BCPE. Po-RD/Al-ZnO MCPE showed 99.25% reproducibility. The synthesized Po-RD/Al-ZnO MCPE is a promising electrode material for the sensor applications. The recovery values clearly validated that the real time monitoring of DOA in pharmaceuticals samples has suitable medium for practical application.

## References

[1] Maziarz W (2019). TiO_2_/SnO_2_ and TiO_2_/CuO thin-film nano-heterostructures as gas sensors. Appl. Surf. Sci..

[2] Gong Y, Wu X, Li X, Wang A, Zhang M, Chen Y (2020). Enhanced acetone sensing properties of Pt@Al-doped ZnO core-shell nanoparticles. Sens. Actuators B Chem..

[3] Bochenkov VE, Sergeev GB (2010). Sensitivity, selectivity, and stability of gas-sensitive metal-oxide nanostructures. Metal Oxide Nanostructures and Their Applications.

[4] Miller DR, Akbar SA, Morris PA (2014). Nanoscale metal oxide-based heterojunctions for gas sensing: A review. Sens. Actuators B Chem..

[5] Kim H-J, Lee J-H (2014). Highly sensitive and selective gas sensors using p-type oxide semiconductors: Overview. Sens. Actuators B Chem..

[6] Shetti NP, Malode SJ, Ilager D, Reddy KR, Shukla SS, Aminabhavi TM (2019). A novel electrochemical sensor for detection of molinate using ZnO nanoparticles loaded carbon electrode. Electroanalysis.

[7] Chang FC, Zhu Z, Luo PY, Wu RJ, Li W (2014). Au@ZnO core–shell structure for gaseous formaldehyde sensing at room temperature. Sens. Actuators B.

[8] Kang SK, Kang DY, Park JW, Kyung RS, Tae GK (2021). Work function-tunable ZnO/Ag/ZnO film as an effective hole injection electrode prepared via nickel doping for thermally activated delayed fluorescence-based flexible blue organic light-emitting diodes. Appl. Surf. Sci..

[9] Shixiang D, Xiaoyan L, Xiaoli J, Qiang H, Yuqi Y, Qiaoji Z, Dunmin L (2020). Core-shell nanostructured ZnO@CoS arrays as advanced electrode materials for high-performance supercapacitors. Electrochim. Acta.

[10] RuiXu KaiYang (2020). YueZang, ZnO/Ag/ZnO multilayer transparent electrode for highly-efficient ITO-Free polymer solar cell. Curr. Appl. Phys..

[11] Kong H, Lee H-Y (2020). High performance flexible transparent conductive electrode based on ZnO/AgOx/ZnO multilayer. Thin Solid Films.

[12] Palmer M, Masikini M, Jiang L-W, Wang J-J, Cummings F, Chowdhury M (2020). Dataset of N-doped CuO:NiO mixed oxide thin film sensor for glucose oxidation. Data Brief..

[13] Hagedorn K, Li W, Liang Q, Dilger S, Noebels M, Wagner MR (2016). Catalytically doped semiconductors for chemical gas sensing: Aerogel-like aluminum-containing zinc oxide materials prepared in the gas phase. Adv. Funct. Mater..

[14] Das S, Girija KG, Debnath AK, Vatsa RK (2021). Enhanced NO_2_ and SO_2_ sensor response under ambient conditions by polyol synthesized Ni doped SnO_2_ nanoparticles. J. Alloys Compd..

[15] Indrajith Naik E, Bhojya Naik HS, Sarvajith MS, Pradeepa E (2021). Co-precipitation synthesis of cobalt doped ZnO nanoparticles: Characterization and their applications for biosensing and antibacterial studies. Inorg. Chem. Commun..

[16] Zhao C, Gong H, Niu G, Wang F (2020). Ultrasensitive SO_2_ sensor for sub-ppm detection using Cu-doped SnO_2_ nanosheet arrays directly grown on chip. Sens. Actuators B Chem..

[17] Singh SK, Dutta D, Dhar A, Das S, Paul MC, Gangopadhyay TK (2019). Detection of ammonia gas molecules in aqueous medium by using nanostructured Ag-doped ZnO thin layer deposited on modified clad optical fiber. Phys. Status Sol..

[18] Hjiri M, Mir LE, Leonardi SG, Pistone A, Maviliad L, Neri G (2014). Al-doped ZnO for highly sensitive CO gas sensors. Sens. Actuators B Chem..

[19] Piovesan JV, Santana ER, Spinelli A (2020). A carbon paste electrode improved with poly (ethylene glycol) for tannic acid surveillance in beer samples. Food Chem..

[20] Saleh TA, Abdur K, Rahim MM (2018). Electrochemical sensor for the determination of ketoconazole based on gold nanoparticles modified carbon paste electrode. J. Mol. Liq..

[21] Rahim AMA, Gaber AAA (2020). Fabrication and characterization of extrinsic electrochemically modified graphite reinforcement carbon paste electrode for selective determination of Cu(II) in trace levels. Appl. Surf. Sci. Adv..

[22] Cariati LSS, Buoro RM (2019). Evaluation of ionic natural deep eutectic solvents (NADES) modified binders towards the chemical properties of carbon paste electrodes. Electrochem. Commun..

[23] Shukla SK, Lavon A, Shmulevich O, Ben-Yoav H (2018). The effect of loading carbon nanotubes onto chitosan films on electrochemical dopamine sensing in the presence of biological interference. Talanta.

[24] Oren T, Birel O, Anık U (2018). Electrochemical determination of dopamine using a novel perylenediimide-derivative modified carbon paste electrode. Anal. Lett..

[25] Fang J, Xie Z, Wallace G, Wang X (2017). Co-deposition of carbon dots and reduced graphene oxide nanosheets on carbon-fiber microelectrode surface for selective detection of dopamine. Appl. Surf. Sci..

[26] Sandoval-Rojas AP, Cortés MT, Hurtado J (2019). Electrochemical synthesis of poly (3,4-ethylenedioxythiophene) doped with a new bis(pyrazolyl)methane disulfonate and its behavior towards dopamine detection. J. Electroanal. Chem..

[27] Ashoka NB, Swamy BEK, Jayadevappa H (2017). Nanorod TiO_2_ sensor for dopamine: A voltammetric study. New J. Chem..

[28] Rashti A, Moncada J, Zhang X, Carrero CA, Oh TS (2019). Thermally grown copper nanowire electrodes modified by electropolymerization. Mater. Chem. Phys..

[29] Islam MR, Rahman M, Farhad SFU, Podder J (2019). Structural, optical and photocatalysis properties of sol–gel deposited Al-doped ZnO thin films. Surf. Interfaces.

[30] Karmakar R, Neogi SK, Banerjee A, Bandyopadhyay S (2012). Appl. Surf. Sci..

[31] Naik EI, Naik HSB, Viswanath R, Kirthan BR, Prabhakara MC (2020). Effect of zirconium doping on the structural, optical, electrochemical and antibacterial properties of ZnO nanoparticles prepared by sol-gel method. Data Collect..

[32] Zamiri R, Singh B, Belsley MS, Ferreira JMF (2014). Structural and dielectric properties of Al-doped ZnO nanostructures. Ceram. Int..

[33] Shashikumara JK, Kumara Swamya BE, Sharma SC (2020). A simple sensing approach for the determination of dopamine by poly (Yellow PX4R) pencil graphite electrode. Chem. Data Collect..

[34] Shashikumara JK, Bhimanagowda K, Kumara Swamya BE, Nagabhushana H, Sharma SC, Lalitha P (2021). Effect of RGO-Y_2_O_3_ and RGO-Y_2_O_3_:Cr^3+^ nanocomposite sensor for dopamine. Sci. Rep..

[35] Pruneanu S, Biris AR, Pogacean F, Socaci C, Coros M, Rosu MC, Watanabe F, Biris AS (2015). The influence of uric and ascorbic acid on the electrochemical detection of dopamine using graphene-modified electrodes. Electrochim. Acta.

[36] Thiagarajan S, Chen SM (2007). Preparation and characterization of Pt Au hybrid film modified electrodes and their use in simultaneous determination of dopamine, ascorbicacid and uric acid. Talanta.

[37] Corona-Avendano S, Ramirez-Silva MT, Palomar-Pardave M, Hernandez-Martinez L, Romero-Romo M, Alarcon-Angeles G (2010). Influence of CTAB on the electrochemical behavior of dopamine and on its analytic determination in the presence of ascorbic acid. J. Appl. Electrochem..

[38] Ali Kamyabi M, Aghajanloo F (2009). Electrocatalytic response of dopamine at a CPE modified with ferrocene. Roat. Chem. Act..

[39] Naik TSSK, Mwaurah MM, KumaraSwamy BE (2018). Fabrication of poly (sudan III) modified carbon paste electrode sensor for dopamine: A voltammetric study. J. Electroanal. Chem..

[40] Goyal RN, Singh SP (2008). Simultaneous voltammetric determination of dopamine and adenosine using a single-walled carbon nano tube—MCPE. Carbon.

[41] Wang Q, Li N, Wang W (2002). Electrocatalytic response of dopamine at a metallothioneins self-assembled gold electrode. Anal. Sci..

[42] Zhu Z, Lining Qu, Guo Y, Zeng Y, Sun W, Haung X (2010). Electrochemical detection of dopamine on a Ni/Al layered double hydroxide modified carbon ionic liquid electrode. Sens. Actuators B.

[43] Kaur B, Pandiyan T, Satpati B, Srivastava R (2013). Simultaneous and sensitive determination of ascorbic acid, dopamine, uric acid, and tryptophan with silver nanoparticles-decorated reduced graphene oxide modified electrode. Colloids Surf. B.

[44] Silva LVD, Silva FAS, Kubota LT, Lopes CB, Lima PR, Costa EO, Junior WP, Goulart MOF (2016). Amperometric sensor based on carbon nanotubes and electropolymerized vanillic acid for simultaneous determination of ascorbic acid, dopamine, and uric acid. J. Solid State Electrochem..

[45] Chih YK, Yang MC (2014). Simultaneous detection of dopamine and ascorbic acid using silver. Taiwan Inst. Chem. Eng..

[46] Liu R, Zeng XB, Liu JC, Luo J, Zheng YY, Liu XY (2016). A glassy carbon electrode modified with an amphiphilic, electroactive and photosensitive polymer and with multi-walled carbon nanotubes for simultaneous determination of dopamine and paracetamol. Microchim. Acta.

[47] Shashikumara JK, Kumara Swamy BE, Madhuchandra HD (2020). Poly (amido black) modified carbon paste electrode sensor for dopamine in the presence of uric acid. Mater. Sci. Energy Technol..

[48] Yang YJ, Li WK (2014). CTAB functionalized graphene oxide/multiwalled carbon nanotube composite modified electrode for the simultaneous determination of ascorbic acid, dopamine, uric acid and nitrite. Sens. Actuators B Chem..

[49] Wang C, Du J, Wang H, Zou C, Jang F, Yang P, Du Y (2014). A facile electrochemicalsensor based on reduced graphene oxide and Au nanoplates modified glassycarbon electrode for simultaneous detection of ascorbic acid, dopamine, anduric acid. Sens. Actuator B Chem.

[50] Yang L, Liu D, Huang JS, You TY (2014). Simultaneous determination of dopamine, ascorbic acid and uric acid at electrochemically reduced graphene oxide modified electrode. Sens. Actuators B Chem..

